# Facilitators of and barriers to labor market participation among people with acquired deafblindness: A scoping review

**DOI:** 10.1371/journal.pone.0345172

**Published:** 2026-03-18

**Authors:** Eline C. M. Heppe, Natascha S. Søndergaard, Emma Klaassen, Michelle Bak, Marleen Smits, Emma Vaillant

**Affiliations:** 1 Royal Kentalis Academy, Utrecht, The Netherlands; 2 Section of Methods and Statistics, Faculty of Behaviour and Movement Sciences, VU Amsterdam, Amsterdam, The Netherlands; International Islamic University Malaysia, MALAYSIA

## Abstract

This scoping review aims to identify factors that facilitate or hinder labor market participation of people with an acquired hearing *and* vision impairment, also called acquired deafblindness (ADB). For people with ADB, participation in society is not a given, and work is a key aspect of societal participation. A literature search was conducted in Web of Science, MEDLINE, and PsycINFO via Ovid. The studies included involved people with ADB, results on labor market participation, and a description of at least one facilitator or barrier. Of 2,548 articles identified, 13 met the inclusion and exclusion criteria. Using the International Classification of Functioning, Disability and Health (ICF) framework, 90 facilitators and 66 barriers were identified. Most facilitators and barriers were identified in the “*Environmental Factors*” domain, while the fewest facilitators were in the “*Body Structures and Body Functions*” and the fewest barriers in “*Personal Factors*.” As more facilitators than barriers were identified, labor market participation could be improved for people with ADB. However, most barriers were found in the “*Services, Systems, and Policies*” subdomain, suggesting vocational rehabilitation services may not be as effective as intended. Further research is needed to explore these barriers and their impact on employment for people with ADB.

## Introduction

Work is a vital aspect of a meaningful and healthy life [1]. Not only does it provide structure in daily life and financial resources, but it has also been found to be positively associated with factors such as wellbeing, socioeconomic status, and life purpose [2,3]. Work is an important part of societal participation and contributes to social inclusion. This is also true for people with disabilities [4]. However, access to the labor market is not a given for people with disabilities [5].

The United Nations Convention on the Rights of Persons with Disabilities (UNCRPD) states that people with disabilities have the right to participate in society and should have the opportunity to earn a living by working in an equal and inclusive work environment [6,7]. Although participation in society is the main goal of clinical rehabilitation and education for people with disabilities, this goal is not always achieved [8]. It is important to focus on work as a means of successful participation for people with disabilities, particularly since finding and maintaining employment remains a challenge for them. Even those who manage to find work may experience difficulties in the workplace, such as inequality in employment conditions [9], stigmatization, social exclusion, and lack of accessibility [10,11]. In addition, employers report concerns about the employment of people with disabilities at various stages of the employment cycle [12]. Employers’ attitudes seem to be an important barrier to the employment of people with disabilities [13].

For people with a hearing *and* vision impairment, also called deafblindness (DB), participation in society can be challenging [14]. People with DB are generally less likely to be employed than those without disabilities, with only 23% employed in Ireland and 29% in the United States, compared to 70% and 75% of the general population, respectively [15]. Moreover, employment rates among people with DB are lower than among those with a single sensory impairment such as a hearing or visual impairment [16].

Within the population of people with DB, three subpopulations can be distinguished: people with *congenital* DB (CDB), people with *acquired* DB, and people with *age-related* DB [17]. In people with *acquired* DB (ADB), the auditory and/or vision impairments arise after language acquisition [17]. Most people in this subpopulation live with the inherited condition of Usher syndrome; they are often born deaf or hard-of-hearing and later develop vision impairment. People with ADB face challenges related to identity development, self-management, and coping with ongoing change and uncertainty [18,19]. Although most people with ADB live independently in the community, often with support, research indicates that they encounter challenges related to employment [20–22]. They face unique difficulties compared to people with other types of disabilities. The combination of vision and hearing loss can lead not only mobility limitations and challenges in performing work tasks, but also to communication difficulties in the workplace, reduced concentration due to fatigue, and decreased work efficiency resulting from inadequate workplace accommodations. Conversely, choosing employment that aligns with the progression of the disability, along with receiving both technical and emotional support from their social network, appears to facilitate employment for people with ADB [22].

Although the above-mentioned challenges and facilitators provide some insight into the labor market participation of people with ADB, the most critical facilitators and barriers remain unclear. To better understand the interaction between the impairment, labor market participation, and personal and environmental factors, this study employed the International Classification of Functioning, Disability and Health (ICF) framework. Developed by the World Health Organization, the ICF framework conceptualizes functioning and disability across the following domains: body functions and body structures, activities and participation, and the contextual domain [23]. Its standardized, internationally recognized structure allows for a systematic identification and categorization of facilitators and barriers at both the individual and environmental levels, making it particularly suitable for analyzing factors related to labor market participation of people with ADB.

This review aimed to identify factors that facilitate or hinder labor market participation of people with ADB, categorized according to the ICF framework. The research question guiding this review was: What are the factors that facilitate or hinder labor market participation among people with ADB? As this research question is exploratory and aims to map the extent and nature of available evidence, a scoping review was conducted.

## Materials and methods

### Search strategy and data sources

A literature search was performed based on the Preferred Reporting Items for Systematic Reviews and Meta-Analysis (PRISMA) statement [24]. To identify all relevant publications, we conducted systematic searches in the bibliographic databases of Web of Science, MEDLINE, and PsycINFO via Ovid from inception to November 6, 2024. Free text search terms were used in all databases. Search terms for subject headings and key terms expressing “deafblindness” and “dual sensory loss” (population) were combined with search terms comprising “work” (outcome). The references in the articles identified were searched for other relevant publications. Duplicate articles were excluded using Zotero 6.0.26 [25]. The search strategy can be found in the supported information: **S1 File** **Search strategy**.

### Operational definitions

An acquired hearing *and* vision impairment (acquired deafblindness; ADB) is considered a combined impairment in which an auditory and/or vision impairment arises after language acquisition [17]. Areas of attention related to care and support of people in this subgroup are: identity development, coping with loss, self-management, and vision for the future [17]. Based on the ICF framework, the individual’s body structures and functions, activities and participation, and contextual factors can promote or hinder labor market participation. Factors that have a positive impact on, or promote labor market participation, are considered facilitators in this study, while factors that have a negative impact on, or hinder labor market participation, are considered barriers.

### Selection process

In a training phase, 11% of the publications (n = 200) were independently screened by two reviewers [EK, EV] on title and abstract to check agreement on, and fine-tune, the inclusion and exclusion criteria. Subsequently, the reviewers screened all potentially relevant titles and abstracts for eligibility, including those screened in the training phase, using the web application Rayyan.ai [26]. Differences in judgment were resolved by discussion between the reviewers and a third reviewer [EH]. All publications identified were read independently by all reviewers [EH, EK, EV] and checked for eligibility.

The following inclusion criteria were applied: (1) data on at least one participant with ADB; (2) the majority of the participants in the study did not have additional cognitive and/or developmental disabilities; (3) most participants included were of working age (as defined by Statistics Netherlands: aged between 15 and 75 years) [27]; (4) at least one facilitator and/or barrier related to labor market participation (including voluntary work); (5) describes an empirical study with either qualitative or quantitative data, including dissertations; (6) written in English, French or Dutch; and (7) published between January 1973 and November 2024. Studies were excluded if: (1) the data was about participants with vision impairment or hearing loss (deaf/hard-of-hearing), but not dual sensory loss; (2) only participants with age-related DB or CDB were included (age-related DB typically manifests after working age and is therefore less relevant to labor market participation, whereas CDB arises before language acquisition and is often accompanied by additional cognitive or developmental disabilities, resulting in different challenges and pathways to labor market participation compared with ADB); (3) they only focused on quality of life, overall participation, and/or social inclusion in daily life and did not describe any facilitators and/or barriers related to labor market participation; (4) they focused on participation in internships, sheltered and/or supported work environments (facilitators and barriers in these supported settings are expected to differ substantially from those in regular labor market environments) or (5) the paper was an editorial, commentary, non-empirical article, book or book chapter, conference abstract, study protocol, literature review, or was not peer-reviewed. No formal quality assessment or bias appraisal was performed, as the aim of this scoping review was to comprehensively map the available evidence rather than to conduct a systematic review. Only peer-reviewed journal articles were included to ensure a basic level of methodological quality, and grey literature was not considered, as it often lacks standardized methodology, peer review, and sufficient detail on study design and data collection

### Data extraction and synthesis

Three reviewers [EK, MB, MS] extracted the data from the articles included using a structured form to document relevant information (author, country, year of publication, population, methodology, and results: barriers/facilitators). This was independently checked by two reviewers [EH, EV]. Two reviewers [EH, EV] coded the barriers and facilitators based on the ICF codes. To allocate correct ICF codes to the data extracted, two reviewers [EH, EV] trained on 17% of the publications included (*n* = 2). Subsequently, both reviewers each independently coded 50% of the data extracted. All allocated ICF codes were discussed by both reviewers [EV, EH] until agreement was reached on all allocated codes. One reviewer [MB] independently checked all the allocated codes. The assigned ICF codes were analyzed using descriptive statistics. The ICF codes were displayed in a frequency table and Sankey chart, categorized in facilitators and barriers.

The review was not registered. The review protocol, data extraction tables and other materials used in the review can be accessed through the researchers. The first draft of the manuscript was written by four authors [EH, NS, MS, EV]. All authors reviewed and commented on earlier drafts and approved the final version of the manuscript.

## Results

### Search results

**Fig 1:**
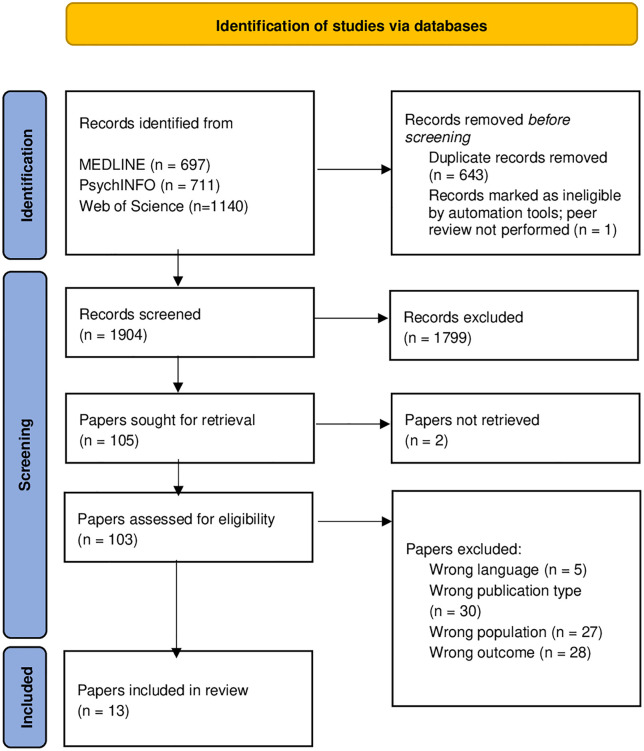
Flowchart of search and selection process.

The literature search generated a total of 2,548 records. After removing 643 duplicates and excluding one record due to the absence of peer review, 1,904 records remained. Following screening and selection based on titles and abstracts, 105 studies remained for full-text reading. The full text could not be retrieved for two of these studies. Ultimately, 13 studies met the inclusion and exclusion criteria, see Fig 1 [17,19,20,27–36]. Excluded studies were either in languages other than English, Dutch or French (*n* = 5), not empirical studies (*n* = 30), included people with only vision impairment, hearing loss (deaf/hard-of-hearing), or CDB (*n* = 27), or did not describe at least one barrier to or facilitator of labor market participation (*n* = 28) (Fig 1).

### Study and participant characteristics

The total number of participants in the 13 studies included was 4,641. Biological sex was not reported for 2,634 participants in three studies and in one study for one dataset [18,29,35–37]. Of the remaining studies, and one of the datasets in one study [29] which had 2,007 participants, 1,049 were male and 958 were female [20,21,28–34]. All studies included data from participants of working age. One study included adolescents aged 13–17 years [32], while another study included adolescents aged 12–17 years and adults aged 18 and older [18].

All studies included data on people with ADB. Two studies included specific professional groups: state directors and business consultants working within a business enterprise program [33] and vocational rehabilitation (VR) agency administrators [36], who reported on people with ADB. One study included a mixed population of people with either ADB or CDB [28]; however, data extracted from this study was specifically related to the participant with ADB. The etiologies of DB were not consistently reported across studies. Nevertheless, five studies [18,20,21,30,31] indicated that all participants were diagnosed with Usher syndrome.

The studies originated from various countries. The majority, 9 out of 13 studies, were from the United States (US) [28–30,32–37]. Two studies were from Sweden [20,21], one study was from Germany [31], and one study was the result of a collaboration between the US and France [18]. Notably, none of the studies were from outside the Western world, specifically from regions such as Asia or Africa. The majority of the studies focused on paid/competitive employment [18,20,21,29–32,34–37]. One study specifically reported on integrated paid community employment [28].

### Facilitators of and barriers to labor market participation

**Fig 2:**
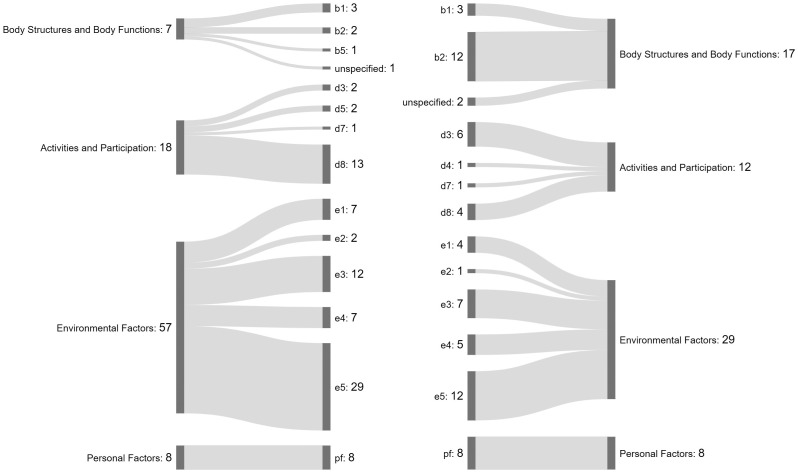
Summary of the facilitators (left) and barriers (right) identified, categorized according to the ICF domains and codes.

The 13 studies reported 90 facilitators and 66 barriers to labor market participation for people with ADB. See Fig 2 for a summary of the facilitators and barriers identified, categorized according to the ICF domains and codes. Most of the reported facilitators and barriers fell within the “*Environmental Factors*” domain. In contrast, the fewest facilitators were reported in the domain of “*Body Structures and Body Functions*,” while the fewest barriers were reported in the domain of “*Personal Factors*.”

Table 1 provides an overview of all ICF codes (with corresponding domains and subdomains) identified in the 13 studies. Within the “*Body Structures and Body Functions*” domain, the studies identified more barriers than facilitators. In particular, multiple studies reported sensory functions such as seeing (b210) and hearing (b230), specifically vision and hearing impairment, as barriers to labor market participation of people with ADB. No specific facilitators related to this domain were consistently reported across the 13 studies.

**Table 1:** Facilitators of and barriers to labor market participation of people with ADB, categorized by ICF codes.

	*Facilitators*		*Barriers*	
ICF code	Amount *n* = 90	Studies *n* = 13	Amount *n* = 66	Studies *n* = 13
**Body Structures and Body Functions**				
Sleep functions (b134)	1	1	1	1
Attention functions (b140)	1	1	1	1
Other specified mental functions (b198)	–	–	1	1
Mental functions, unspecified (b199)	1	1	–	–
Sensory functions and pain: Seeing functions (b210)	1	1	6	6
Sensory functions and pain: Hearing functions (b230)	1	1	6	6
Functions of the digestive, metabolic, and endocrine systems: Weight maintenance functions (b530)	1	1	–	–
Unspecified: Additional disabilities	1	1	2	2
*Total amount of facilitators and barriers in the Body Structures and Body functions domain*	7		17	
**Activity and Participation**				
***Communication***				
Communicating – receiving: Communicating with – receiving – spoken messages (d310)	–	–	1	1
Communicating – receiving, other specified and unspecified (d329)	–	–	1	1
Communication – producing, other specified and unspecified (d349)	–	–	1	1
Conversation and use of communication devices and techniques: Using communication devices and techniques (d360)	2	1	1	1
Communication, unspecified (d399)	–	–	2	1
***Mobility***				
Moving around using transportation, other specified and unspecified (d489)	–	–	1	1
***Self-care***				
Looking after one’s health (d570)	1	1	–	–
Other specified self-care (d598), i.e., work-life balance	1	1	–	–
***Interpersonal interactions and relationships***				
General interpersonal interactions: Complex interpersonal interactions, other specified and unspecified (d720), i.e., not disclosing disability	1	1	–	–
Particular interpersonal relationships: Formal relationships (d740)	–	–	1	1
***Major life areas***				
Education: Vocational training (d825)	3	1	2	2
Education: Higher education (d830)	4	3	1	1
Education: Education, other specified and unspecified (d839)	1	1	–	–
Work and employment: Acquiring, keeping, and terminating a job (d845)	2	2	1	1
Work and employment: Non-remunerative employment (d855)	1	1	–	–
Work and employment, other specified and unspecified (d859)	2	2	–	–
*Total amount of facilitators and barriers in the Activity and Participation domain*	18		12	
**Environmental Factors**				
***Products and technology***				
Products and technology for communication (e125)	4	3	2	2
Products and technology for employment (e135)	3	3	2	2
***Natural environment and human-made changes to environment***				
Natural environment and human-made changes to environment, unspecified (e299)	2	2	1	1
***Support and relationships***				
Immediate family (e310)	1	1	1	1
Extended family (e315)	–	–	1	1
Acquaintances, peers, colleagues, neighbors, and community members (e325)	7	5	2	1
People in positions of authority (e330)	1	1	1	1
Strangers (e345)	1	1	–	–
Other professionals (e360)	2	1	2	1
***Attitudes***				
Individual attitudes of immediate family members (e410)	2	1	–	–
Individual attitudes of acquaintances, peers, colleagues, neighbors, and community members (e425)	1	1	2	2
Individual attitudes of people in positions of authority (e430)	1	1	1	1
Individual attitudes of other professionals (e455)	2	2	2	1
Other specified attitudes (e498), i.e., organizational attitude	1	1	–	–
***Services, systems, and policies***				
Education and training services, systems, and policies (e585)	5	4	1	1
Social security services, systems, and policies (e570)	–	–	4	2
Labor and employment services, systems, and policies (e590)	23	10	7	3
Other specified services, systems, and policies (e598)	1	1	–	–
*Total amount of facilitators and barriers in the Environmental Factors domain*	57		29	
**Personal Factors**				
Age	3	3	2	2
Biological sex	1	1	3	2
Age of diagnosis	1	1	1	1
Personal attitudes	1	1	2	1
Self-reliance	2	1	–	–
*Total amount of facilitators and barriers in the Personal Factors domain*	8		8	

In the “*Activity and Participation*” domain, more facilitators than barriers were identified. Most facilitators were found within the “*Major Life Area*” subdomain, with educational background reported in multiple studies as a facilitator of labor market participation of people with ADB. Higher education (d830) – which includes universities and colleges – was the most frequently reported facilitator. This was followed by vocational training (d825), which involves participating in vocational programs and acquiring skills necessary for employment. However, this was not consistently reported as a facilitator across the studies. The most barriers were reported in the “*Communication*” subdomain of “*Activity and Participation*,” although these barriers were not consistently reported across the studies.

The domain with the highest number of reported facilitators and barriers was “*Environmental Factors*,” where more facilitators than barriers were reported. Most facilitators were reported in the “*Services, Systems, and Policies*” subdomain, with labor and employment services, systems, and policies (e590) – which encompass services, systems, and policies aimed at assisting unemployed people in finding suitable work or support employed people seeking advancement – frequently reported in multiple studies as a facilitator of labor market participation of people with ADB. Within this subdomain, education and training services, systems, and policies (e585) were also reported in multiple studies as a facilitator of labor market participation of people with ADB.

Other commonly reported facilitators within the domain of “*Environmental Factors*” were support and relationships with acquaintances, peers, colleagues, neighbors, and community members (e325); and products and technology for communication (e125) and employment (e135). The most barriers were reported in the “*Services, Systems, and Policies*” subdomain, with labor and employment services, systems, and policies (e590) also frequently reported as a barrier to labor market participation of people with ADB in multiple studies.

In the “*Personal Factors*” domain, an equal number of barriers and facilitators were reported. This domain covers individual characteristics not directly related to the health condition and is not specified by ICF codes. Within this domain, age was reported as a facilitator in multiple studies. Most barriers within this domain were not consistently reported across the studies.

### Body structures and body functions

#### Facilitators.

Three out of the 13 studies reported factors related to “*Body Structures and Body Functions*” that could act as facilitators of labor market participation of people with ADB [20,31,32]. One study reported that people with Usher syndrome experienced less stress related to work overload, social overload, and success pressure compared to a control group of people without disabilities, which could act as a facilitator of employment [31]. Another study indicated that fewer mental and/or physical health problems could facilitate employment of people with ADB [20]. A third study reported that when considering other factors, the number of additional disabilities was not always a barrier to employment. The authors explained that youth with multiple disabilities who secure a job and receive adequate support are more likely to remain employed compared to youth with fewer multiple disabilities [32].

#### Barriers.

Eight out of 13 studies reported factors related to “*Body Structures and Body Functions*” that could act as barriers to labor market participation of people with ADB [18,20,21,29–31,34,37]. Six of these seven studies found that ADB itself negatively impacted labor market participation and therefore could be seen as a barrier to employment [18,21,29–31,37], although in one study [37], the relation between ADB and leaving the labor force due to a disability, was not significant after controlling for sex, ethnicity, number of health conditions and self-reported health. Studies on people with Usher syndrome reported they experience difficulties with career choices, a need for continuous task modifications, more stress related to being overwhelmed by work, social tensions, social isolation, chronic worry, and decreased productivity due to the progressive nature of the syndrome. Three of the eight studies reported that additional mental and/or physical health problems and disabilities also acted as barriers to labor market participation of people with ADB [20,21,34]

### Activity and participation

#### Facilitators.

Nine out of the 13 studies reported factors related to “*Activity and Participation*” that could act as facilitators of labor market participation of people with ADB [20,21,28–30,32–35]. Facilitators were identified within four of the nine subdomains of “*Activity and Participation*”: “*Communication*,” “*Self-care*,” “*Interpersonal Interactions and Relationships*,” and “*Major Life Areas*.” Most of the facilitators were reported within the subdomain of “*Major Life Areas*.” Within this subdomain, four of the nine studies reported that educational background could facilitate labor market participation of people with ADB [29,32,34,35]. Specifically, obtaining higher levels of education (university, college degree) [29,34,35] or receiving vocational or career education [32,34] were reported to facilitate employment. Five of these nine studies reported that work and employment background could also facilitate labor market participation of people with ADB [28–30,32,34]. Particularly, having experience from previous employment (volunteer work or high school jobs) was reported as a facilitator [28,32,34]. Other work-related factors, such as having the ability to change jobs and taking the initiative to reach out to employers, were also reported as facilitators of labor market participation of people with ADB [29,30].

Four of the nine studies reported additional facilitators of labor market participation of people with ADB within the subdomains of “*Communication*,” “*Self-care*,” and “*Interpersonal Interactions and Relationships*.” One study reported that using communication devices and techniques for the purpose of communicating could facilitate employment [33]. Two studies reported that maintaining good self-care, such as being in good physical health and achieving a good work-life balance, could facilitate employment [20,21]. Additionally, one study reported that not disclosing the disability itself during the job application process could be a facilitator of labor market participation of people with ADB [30].

#### Barriers.

Four out of the 13 studies reported factors related to “*Activity and Participation*” that could act as barriers to labor market participation of people with ADB [28,29,33,35]. Barriers were identified within four of the nine subdomains of “*Activity and Participation*”: “*Communication*,” “*Mobility*,” “*Interpersonal Interactions and Relationships*,” and “*Major Life Areas*.” Most of the barriers were reported within the subdomains of “*Communication*” and “*Major Life Areas*.” Not being able to communicate effectively with colleagues, staff, and customers was reported several times as a barrier to labor market participation of people with ADB, but only in one study [33]. Within the subdomain of “*Major Life Areas*,” two studies reported that educational background could act as a barrier to employment [29,35]. Specifically, not having a college degree and lack of training for a job were reported as barriers to employment. In terms of work and employment background, being unable to change jobs was reported as a barrier to employment [29]. The same study also reported that transportation problems related to ADB could act as a barrier to employment, as it is often more difficult for people with ADB to find transport to their workplace [29]. Finally, one study reported that the inability to socially integrate into an organization due to the disability was also a barrier to labor market participation of people with ADB [28].

### Environmental factors

#### Facilitators.

Eleven out of 13 studies reported “*Environmental Factors*” that could act as facilitators of labor market participation of people with ADB [20,21,28–36]. Facilitators were identified within all five subdomains of “*Environmental Factors*”: “*Products and Technology*,” “*Natural Environment and Human-made Changes to Environment*,” “*Support and Relationships*,” “*Attitudes*,” and “*Services, Systems, and Policies*.”

Most of the facilitators of labor market participation of people with ADB were reported within the subdomain of “*Services, Systems, and Policies*.” Within this subdomain, 10 of the 11 studies reported that labor and employment services, systems, and policies could facilitate employment [20,28–36]. Several types of employment services were described in these studies, with receiving help from vocational rehabilitation (VR) counsellors or employment counsellors being the most frequently reported program facilitating employment for people with ADB [20,29–32,34,35]. The studies reported that these counsellors provided support in several phases of the employment process, such as finding employment by helping to reach out to employers, practicing job interviews, and keeping employment through job skill (re)training for people with ADB.

One study also described several skills these counsellors should have, such as knowledge of ADB, the ability to communicate with people with ADB (e.g., using sign language), and a belief in the employability of people with ADB [30]. This study also emphasized that people with ADB should be involved in the process of planning and goal setting with the counsellors. Other services and policies were also reported in the studies, including: employing people with ADB alongside people with other disabilities and people without disabilities; restructuring jobs to make them more accessible to people with ADB; implementing early interventions that include work-promoting activities and skills; and creating guidelines for employers on hiring people with ADB, including procedures for incorporating interpreters into the workplace, providing information on communication strategies, and hiring personnel to support people with ADB on the job [20,28,33,36].

Education and training services, systems, and policies were also reported as facilitators of labor market participation of people with ADB [28,29,32,33]. These services include skill training for people with ADB and their co-workers to facilitate effective communication, such as (tactile) sign language, training VR counsellors, colleagues, and staff to improve their understanding of ADB, and educating parents about employment possibilities for their child with ADB. Additionally, one study reported that employment programs for people with ADB can benefit from partnerships with external organizations specializing in ADB, as these collaborations can enhance resources and support for their employees with ADB [33].

Five out of 11 studies reported additional facilitators of labor market participation of people with ADB within the subdomain of “*Support and Relationships*” [21,28,30,32,33]. All five studies reported that support and relationships with acquaintances, peers, colleagues, neighbors, and community members could facilitate employment of people with ADB. This type of support included support from colleagues with knowledge of sign language, and support of other peers with ADB (experts-by-experience). Additionally, support and relationships with immediate family members (such as parents), people in positions of authority (such as managers), and other professionals (such as interpreters), were reported as facilitators of labor market participation of people with ADB [21,32,33].

Five out of 11 studies reported additional facilitators of labor market participation of people with ADB within the subdomain of “*Attitudes*” [21,29,30,32,33]. Two studies reported that the positive attitudes of professionals, such as VR counsellors and state directors, regarding the employment potential and success of people with ADB, could facilitate labor market participation by this population [30,33]. One study reported that the attitudes of immediate family members, particularly the expectations of parents regarding the employment possibilities for their child, could also facilitate employment [32]. Another study reported that the attitudes of acquaintances, peers, colleagues, neighbors, and community members, as well as people in positions of authority, such as managers, who view people with ADB as competent employees, could facilitate their employment [21]. Additionally, one study reported that it could be beneficial for people with ADB to work in rehabilitation organizations for people with vision and/or hearing impairment, as these organizations might have more favorable attitudes toward making accommodations related to ADB [29].

Four out of 11 studies reported additional facilitators of labor market participation of people with ADB within the subdomain of “*Products and Technology*” [28,29,31,33]. Within this subdomain, three studies [28,29,33] reported that communication products and technology could facilitate employment of people with ADB. Additionally, three studies [29,31,33] reported that products and technology for employment could facilitate employment of people with ADB. These products included Assistive Technology (AT) and Augmentative and Alternative Communication (AAC), such as braille and speech devices, sound amplifiers, special lightning, sign language, and laptops, which all enable people with ADB to communicate with colleagues and perform job-related tasks.

Two out of 11 studies reported additional facilitators within the subdomain of “*Natural Environment and Human-made Changes to Environment*” [29,31]. Both studies reported that workplace accommodations related to ADB could facilitate employment. However, no specific accommodations were reported.

#### Barriers.

Nine out of 13 studies reported “*Environmental Factors*” that could act as barriers to labor market participation of people with ADB [18,20,28–30,33–36]. Barriers were identified within all five subdomains of “*Environmental Factors*”: “*Products and Technology*,” “*Natural Environment and Human-made Changes to Environment*,” “*Support and Relationships*,” “*Attitudes*,” and “*Services, Systems, and Policies*.”

Most of the barriers to labor market participation of people with ADB were reported within the subdomain of “*Services, Systems, and Policies*,” thus mirroring the facilitators. Within this subdomain, three out of the nine studies reported that labor and employment services, systems, and policies could act as barriers to employment [30,34,36]. Various types of support services were described that could not facilitate employment of people with ADB, such as VR counsellors with low employment expectations for people with ADB and programs only given short-term support [30,34]. Additionally, services that are not specialized in ADB or do not collaborate with other sensory disability agencies could be a barrier to employment of people with ADB [34,36]. Lack of assistance from a VR counsellor, inadequate support from a VR counsellor, or high turnover rates of VR counsellors were also reported as barriers to employment [30].

Other services, such as social security services, systems, and policies, were also reported as barriers to employment of people with ADB. Two out of nine studies reported that receiving social security benefits or a disability pension were barriers to employment of people with ADB, as this type of support could reduce motivation to return to work and lead to a loss of confidence or feelings of worthlessness [20,34]. Additionally, one study reported that education and training services, systems, and policies could be barriers to labor market participation of people with ADB, particularly when education and training services do not address specific needs of people with ADB [35].

Three out of nine studies reported additional barriers to labor market participation of people with ADB within the subdomain of “*Attitudes*” [18,29,30]. Two studies reported that the attitudes of acquaintances, peers, colleagues, neighbors, and community members could be barriers to employment, reporting that particularly negative reactions and discrimination by colleagues could hinder employment [18,29]. One study reported that the attitudes of other professionals, such as VR counsellors or rehabilitation professionals, could hinder employment of people with ADB, particularly when they hold low expectations of their employment potential [30]. Another study reported that attitudes of those in authority, such as managers or employers, can also be a barrier, especially when they discriminate against people with ADB or pressure them to resign [29].

Three out of nine studies reported additional barriers to labor market participation of people with ADB within the subdomain of “*Products and Technology*” [28,29,33]. Two studies reported that communication products and technology could be barriers to employment, as some communication systems require close proximity, touch, or fluency in sign language, which may limit the ability to socially integrate in the workplace [28,33]. Additionally, two studies reported that employment-related products and technology could pose barriers, particularly when people with ADB lack experience with these technologies or when the products become outdated or require updates, making them unsuitable for performing work-related tasks [29,33].

Two out of nine studies reported additional barriers to labor market participation of people with ADB within the subdomain of “*Support and relationships*” [29,33]. One study reported that support from and relationships with people in positions of authority, such as managers and employers, could be barriers to employment, particularly if these individuals force people with ADB into retirement [29]. Additionally, another study reported that support from and relationships with colleagues and professionals could pose barriers to employment. Specifically, the reliance on a third party, such as colleagues who know sign language, or the availability of interpreters, for communication with people with ADB was reported as a barrier to their employment [33].

One out of nine studies reported additional barriers to labor market participation of people with DB within the subdomain of “*Natural Environment and Human-made Changes to Environment*” [29]. This study reported that the need for workplace accommodations related to ADB, and the lack of such accommodations, could be a barrier to employment of people with ADB.

### Personal factors

#### Facilitators.

Four out of the 13 studies reported “*Personal Factors*” that could act as facilitators of labor market participation of people with ADB [20,31,33,34]. Three of these four studies reported that age was a facilitator of employment [20,31,34]. One study reported that being older facilitated employment of people with ADB, as work-related stress tends to decrease with age [31]. However, two studies reported that being younger was a facilitator of employment, as younger people with ADB are more often employed [20,34]. One of these studies also reported that receiving a diagnosis of ADB at a younger age could facilitate employment, as it may positively impact career planning [20]. One study reported biological sex as a facilitator of employment for people with ADB; specifically, being a male was reported as a facilitator [31]. Another study reported that personal attitudes of people with ADB toward their ability to work could also be a facilitator of employment, with the belief that having ADB is not a barrier to employment being a positive factor for employment [33]. Additionally, one study reported that self-reliance, where people with ADB view themselves as their own primary source of support, facilitates labor market participation of people with ADB [34].

#### Barriers.

Four out of 13 studies reported “*Personal Factors*” that could act as barriers to labor market participation of people with ADB [20,29,31,34]. One study reported that being younger was a barrier to employment of people with ADB, as work-related stress tends to increase with age [31]. However, another study reported that being older was also a barrier, as older people with ADB are less often employed [20]. This study also noted that receiving a diagnosis of ADB at an older age can hinder labor market participation of people with ADB. This is because it may lead to challenges such as the chosen profession becoming unsustainable due to the progressive nature of the disability, delays in vocational training, and the potential for extended sick leave following the diagnoses, which can complicate the process of returning to work. In two studies, biological sex was reported as a barrier to employment for people with ADB; specifically, being a female was reported as a barrier [31,34]. Additionally, one study reported that personal attitudes of people with ADB toward their ability to work can also serve as a barrier. It reported that the belief that having ADB poses a barrier to employment of people with ADB is itself a barrier [33].

## Discussion

By using the ICF framework, we identified 156 factors that facilitate (*n* = 90) and hinder (*n* = 66) labor market participation of people with ADB. Overall, more facilitators were identified than barriers. The majority of the facilitators and barriers were found in the “*Environmental Factors*” domain, highlighting the importance of the environment in which people with ADB operate. As indicated by the studies, the environment can either support or hinder employment success. In contrast, the “*Body Structures and Body Functions*” domain had the fewest facilitating factors and “*Personal Factors*” the fewest hindering factors. This underscores the notion that labor market participation of people with ADB is not solely dependent on individual characteristics and skills, which are often primarily supported and enhanced in rehabilitation and education services for people with ADB, but may also rely on a supportive environment.

The review identified more facilitators than barriers. This suggests that employment of people with ADB could be supported in multiple ways, potentially leading to higher labor market participation. However, the studies also indicated that finding employment remains a challenge for people with ADB. The “*Services, Systems, and Policies*” subdomain was particularly ambiguous, serving as both a facilitator of and a barrier to labor market participation of people with ADB. Specifically, services such as vocational services (VR) could be beneficial when tailored to the individual’s needs but can become a barrier when they are not. This underscores the importance of high-quality, personalized support services in enhancing the employability of people with ADB. This is supported by studies showing the success of specialized VR services tailored to specific disabilities [38–42].

Furthermore, the review showed that support from and relationships with family members, colleagues, professionals, and peers (e.g., other experts-by-experience) are important facilitators of labor market participation of people with ADB. Research on typically developing adults also showed that parental support remains important into adulthood [43] and peer relations can bolster resilience against stress [44]. The value of peer support, particularly from peers with ADB, was further highlighted in a recent study of people with Usher syndrome [45]. These findings, combined with the additional findings from this current study, which showed that the attitudes of family members, colleagues, professionals, and peers can facilitate employment, underscore the need for enhancing and supporting the social networks of people with ADB. Based on the studies included in our review, it seems important for people with ADB to not only have a broad social network but also that these networks are supportive, that they recognize the employment potential of people with ADB, and that they facilitate opportunities for their professional development. This approach may increase the likelihood of finding and maintaining employment by building a strong social safety net.

Another crucial finding was the importance of education, particularly higher education and vocational training, in facilitating labor market participation of people with ADB. This is consistent with previous research, which also demonstrated that higher education levels facilitate employment of people with vision impairments [46]. It should be noted that higher levels of education alone are not sufficient. Gaining early work experience while still at school is also important for labor market participation. For people with ADB, integrating work experience into the educational process can be particularly beneficial. Therefore, educational systems supporting people with ADB should not only focus on academic achievement but also encourage employability and work-related experiences [32].

It is not surprising that the impairment itself (ADB) was frequently identified as a barrier in this review, given the challenges to employability for people with combined hearing and vision impairment. Consequently, we might have expected to find more barriers to than facilitators of labor market participation of people with ADB. While progress is needed to enhance labor market participation of people with ADB, there seem to be many facilitators across all ICF domains of functioning that can be explored when it comes to supporting employment of people with ADB. Specifically, we anticipated communication would be more frequently identified as a barrier, given the known communication difficulties faced by people with DB [17,47,48] and its importance in employment [49]. However, this was not the case. Instead, factors such as workplace accommodations, assistive technologies, and social support were highlighted as facilitators, suggesting communication challenges may be mitigated through these means. This indicates that there are significant opportunities to support employment by focusing on these facilitators.

This review revealed the limited research available on facilitators of and barriers to labor market participation of people with ADB. Many factors identified as facilitators were also reported as barriers, particularly within the “*Services, Systems, and Policies*” subdomain, and similar dual roles were observed in other (sub)domains as well. For example, while alternative communication methods and assistive technologies can sometimes be perceived as barriers by colleagues and employers, their potential as facilitators can be maximized through appropriate training, clear communication, and supportive workplace policies [50–52].The study findings suggest that, although facilitators were more frequently identified in this review, further research is needed from different perspectives, such as those of people with ADB, employers, colleagues, and professionals, to better understand how factors can facilitate or hinder labor market participation of people with ADB.

### Strengths and limitations

One key strength of this review is that almost all of the studies reported on the facilitators and barriers from the perspective of people with ADB themselves. This focus provides a comprehensive understanding of the challenges and support that this population experience from their perspectives. Additionally, this review is notable for being the first extensive review that offers a detailed description of the facilitators and barriers identified in the literature. The inclusion of studies from various countries – such as the US, Sweden, Germany, and France – adds valuable international context and helps to highlight how employment of people with ADB is experienced in different countries.

This latter strength also presents a limitation to the study. This review was limited to studies from Western countries, reflecting the available research, and therefore cannot provide insight into the facilitators and barriers faced by people with ADB in non-Western countries. Labor market structures, social policies, workplace accommodations, and societal attitudes toward disability vary across regions globally, which may influence both the presence and impact of facilitators and barriers. In non-Western contexts, limited access to vocational training, fewer legal employment protections, or reduced availability of workplace accommodations could reduce the impact of facilitators in Western contexts. Conversely, barriers such as rigid workplaces hierarchies, lower awareness of disability rights, or heightened social stigma may be pronounced, potentially altering the relative importance of different facilitators and barriers. This geographical limitation therefore constrains our understanding of how contextual and cultural factors might shape employment outcomes for people with ADB globally. Additionally, the relatively small number of studies limited the depth and robustness of the conclusions. Most facilitators and barriers were identified in only one study, with some studies focusing primarily on overall quality of life or specific conditions such as Usher syndrome, limiting the comparability and generalizability of the findings to the broader population of people with ADB.

Furthermore, while this review incorporated a range of perspectives from people with ADB, it did not include a comprehensive overview of the perspectives of employers, colleagues, and professionals such as VR counsellors in relation to labor market participation of people with ADB. Additionally, variation in national policies and structures, particularly within the domain of “*Services, Systems, and Policies*,” may impact the ability to draw universal conclusions about which services might be effective. Finally, while most articles in this review were published within the last ten years, two older articles from 1991 and 2008 were also included. Due to economic shifts and changes in societal context over time, these may not fully align with the current labor market status of people with ADB. These limitations underscore the need for further research, exploring the facilitators of and barriers to labor market participation of people with ADB, drawing from diverse perspectives (including those of experts-by-experience, employers, and professionals) and reflecting the current economic context in various countries.

### Implications for practice

Based on the studies included and considering the limitations of this review, we propose three recommendations for practice. First, it may be beneficial for people with ADB to have access to vocational rehabilitation (VR) counsellors who possess specialized knowledge of ADB. These counsellors should be able to tailor their services to support the unique challenges and needs of people with ADB in seeking and maintaining employment. Alternatively, it may be advantageous for VR counsellors to collaborate closely with ADB specialists to enhance the support provided to this population. This approach would enable counsellors to directly address the specific challenges associated with ADB, including understanding and implementing workplace accommodations that facilitate employability. Combining specialized expertise with consistent professional collaboration, as described by McDonnall and Cmar [36], may provide a structured approach that integrates both expert knowledge and coordinated service delivery, potentially enhancing support for people with ADB.

Second, we recommend that when workplace accommodations are made for people with ADB, particular attention should be given to supporting others in the work environment, especially employers and colleagues. Fostering an inclusive workplace is essential for enhancing the labor market participation of people with ADB. Hierholzer et al. [33] highlighted the importance of improving communication between employees with DB and their trainers. They suggested, for example, providing opportunities for trainers and counsellors to learn (tactile) sign language and expanding access to interpreters, including employing interpreters as staff members. These measures are suggested to support mutual understanding, facilitate communication, and enhance inclusion within the workplace.

Finally, as indicated by multiple studies, obtaining higher education is highly beneficial in ensuring future employment opportunities of people with ADB [29,32,34,35]. This education is particularly valuable when it incorporates work experience, as this provides people with ADB the chance to gain relevant, hands-on experience, which can be instrumental in their transition to the labor market.

## Conclusion

In this scoping review, we addressed the research question: What are the factors that facilitate or hinder labor market participation among people with ADB? A total of 13 research articles met the inclusion criteria, and using the ICF framework, we identified 90 facilitators and 66 barriers. Most of these were found in the “*Environmental Factors*” domain, demonstrating the importance of the environment of people with ADB with respect to their employment outcomes. Future research should further investigate the impact of various facilitators and barriers from different perspectives across multiple countries on labor market participation of people with ADB. Finally, further exploration of the impact of services such as VR counsellors – in relation to supporting people with ADB and their environment with the aim of enhancing employment outcomes – is needed.

## Supporting information

S1 File Search strategy. (PDF)

S2 File PRISMA-ScR-fillable-checklist-1. (DOCX)

S1 Data Data extractie Tabel 1. (PDF)

S2 Data Data extractie Tabel 2. (PDF)

S3 Data Data extractie Tabel 3. (PDF)

S4 Data Data extractie Tabel 4. (PDF)
