# Supplementary material for: Facilitators of and barriers to labor market participation among people with acquired deafblindness: A scoping review
Source: PLoS One. 2026 Mar 18;21(3):e0345172. doi: 10.1371/journal.pone.0345172 (PMC12998870; doi:10.1371/journal.pone.0345172)
Supplement: S1 File — (PDF) [file pone.0345172.s001.pdf]

S1 File. Search strategy

1	("Usher* syndrome*" or "Graefe-Usher* syndrome*" or "Hallgren* syndrome*" or "deafness retinitis pigmentosa syndrome*" or "dystrophia retinae pigmentosa dysostosis syndrome*" or "retinitis pigmentosa deafness syndrome*").mp.
2	(deafblind* or "deaf-blind*" or "dual sensory loss" or "dual sensory impair*" or "dual sensory disab*" or "dual sensory disorder*" or "dual sensory dysfunction*" or "combined sensory loss" or "combined sensory impair*" or "combined sensory disab*" or "combined sensory disorder*" or "combined sensory dysfunction*").mp.
3	(deaf* or "hard of hearing" or DHH or "loss of hearing" or "aural* handicap*" or "aural* disab*" or "aural* disorder* or aural* dysfunction*" or "hearing impair*" or "hearing disab*" or "hearing loss" or "hearing disorder*" or "auditory impair*" or "auditory disab*" or "auditory loss" or "auditory disorder*" or "auditory dysfunction*").mp.
4	(blind* or "vision loss" or "visual impair*" or "visual disab*" or "visual loss" or "visual disorder*" or "visual dysfunction*" or "visual* handicap*" or "partial* sight*" or "partial vision" or "low vision" or "balint* syndrom*" or "hemianopia" or "functional visual symptom*").mp.
5	1 or 2 or (3 and 4) (=population)
6	(work* or "job" or "jobs" or profess* or vocation* or career* or employ* or unemploy* or "labour" or "labor" or occupation*).mp. (=theme)
7	5 and 6 (=population and theme)
