# Supplementary material for: Facilitators of and barriers to labor market participation among people with acquired deafblindness: A scoping review
Source: PLoS One. 2026 Mar 18;21(3):e0345172. doi: 10.1371/journal.pone.0345172 (PMC12998870; doi:10.1371/journal.pone.0345172)
Supplement: S1 Data — (PDF) [file pone.0345172.s003.pdf]

	Facilitators		*Barriers*	
	Amount	Studies	Amount	Studies
	n = 90	*n* = 13	*n* = 66	*n* = 13
**Body structures and body functions**				
Sleep functions (b134)	1	1	1	1
Attention functions (b140)	1	1	1	1
Other specified mental functions (b198)	-	-	1	1
Mental functions, unspecified (b199)	1	1	-	-
Sensory functions and pain: Seeing functions (b210)	1	1	6	6
Sensory functions and pain: Hearing functions (b230)	1	1	6	6
Functions of the digestive, metabolic and endocrine systems: Weight maintenance functions (b530)	1	1	-	-
Unspecified: Additional disabilities	1	1	2	2
**Activity and participation**				
Communication				
Communicating – receiving: Communicating with – receiving – spoken messages (d310)	-	-	1	1
Communicating – receiving, other specified and unspecified (d329)	-	-	1	1
Communication – producing, other specified and unspecified (d349)	-	-	1	1
Conversation and use of communication devices and techniques: Using communication devices and techniques (d360)	2	1	1	1
Communication, unspecified (d399)	-	-	2	1
Mobility				
Moving around using transportation, other specified and unspecified (d489)	-	-	1	1
Self-care				
Looking after one's health (d570)	1	1	-	-
Other specified self-care (d598), i.e. work-life balance	1	1	-	-
Interpersonal interactions and relationships				
General interpersonal interactions: Complex interpersonal interactions, other specified and unspecified (d720), i.e. not disclosing disability	1	1	-	-
Particular interpersonal relationships: Formal relationships (d740)	-	-	1	1
Major life areas				
Education: Vocational training (d825)	3	1	2	2
Education: Higher education (d830)	4	3	1	1
Education: Education, other specified and unspecified (d839)	1	1	-	-
Work and employment: Acquiring, keeping and terminating a job (d845)	2	2	1	1
Work and employment: Non-remunerative employment (d855)	1	1	-	-
Work and employment, other specified and unspecified (d859)	2	2	-	-
**Environmental factors**				
Products and technology				
Products and technology for communication (e125)	4	3	2	2
Products and technology for employment (e135)	3	3	2	2
Natural environment and human-made changes to environment				
Natural environment and human-made changes to environment, unspecified (e299)	2	2	1	1
Support and relationships				
Immediate family (e310)	1	1	1	1
Extended family (e315)	-	-	1	1
Acquaintances, peers, colleagues, neighbours and community members (e325)	7	5	2	1
People in positions of authority (e330)	1	1	1	1
Strangers (e345)	1	1	-	-
Other professionals (e360)	2	1	2	1
Attitudes				
Individual attitudes of immediate family members (e410)	2	1	-	-
Individual attitudes of acquaintances, peers, colleagues, neighbours and community members (e425)	1	1	2	2
Individual attitudes of people in positions of authority (e430)	1	1	1	1
Individual attitudes of other professionals (e455)	2	2	2	1
Other specified attitudes (e498), i.e. organizational attitude	1	1	-	-
Services, systems and policies				
Education and training services, systems and policies (e585)	5	4	1	1
Social security services, systems and policies (e570)	-	-	4	2
Labour and employment services, systems and policies (e590)	23	10	7	3
Other specified services, systems and policies (e598)	1	1	-	-
**Personal factors**				
Age	3	3	2	2
Gender	1	1	3	2
Age of diagnosis	1	1	1	1
Personal attitudes	1	1	2	1
Self-reliance	2	1	-	-
