# Supplementary material for: Facilitators of and barriers to labor market participation among people with acquired deafblindness: A scoping review
Source: PLoS One. 2026 Mar 18;21(3):e0345172. doi: 10.1371/journal.pone.0345172 (PMC12998870; doi:10.1371/journal.pone.0345172)
Supplement: S2 Data — (PDF) [file pone.0345172.s004.pdf]

1. Body structures and body functions

1.1.1. Facilitators

Study 4:

- People with Usher syndrome experience less stress on the work overload and success pressure (b210 + b230).
  Facilitator from the perspective of person with DB.

Study 5:

- Sleep and concentration: however, the disability pension group reported a significantly higher degree of sleep and concentration problems compared to the working group. [oftewel: werkenden hebben minder slaap- en concentratieproblemen] (b134 + b140).
  Facilitator from the perspective of persons with DB
- Psychological health/mental health: person in the working group had better general psychological health (b199).
  Facilitator from the perspective of person with DB.
- Physical health: persons in the working group had better physical health: less overweight, better at running short distance (b530 + d570).
  Facilitator from the perspective of person with DB.

Study 6:

- Number of additional disabilities: number of disabilities other than the youths primary disability reported by the parent. Additional disabilities is associated with continuous employment. The odds of continuous employment increased by 1.43 with each additional disability. The most commonly reported additional disabilities were health impairment (30.2%, SE = 3.97), physical or orthopedic impairment (27.8%, SE = 4.31), and speech disorder (20.4%, SE = 3.35) (BODY FUNCTIONS, unspecified: additional disabilities).
  Facilitator from the perspective of multiple groups.

1.1.2. Barriers

Study 2:

- Having sensory loss: the experience of dual sensory loss had a significant negative effect on employment (b210 + b230).
  Barrier from the perspective of person with DB.

Study 3:

- Usher syndrome: their choice of a profession or their progress in that profession had been modified or otherwise impacted by the Usher syndrome (b210 + b230).
  Barrier from the perspective of person with DB.

Study 4:

- People with Usher syndrome experience more stress on the scales being overwhelmed with work (b210 + b230).
  Barrier from the perspective of persons with DB.

Study 5:

- Sleep and concentration: however, the disability pension group reported a significantly higher degree of sleep and concentration problems compared to the working group (b134 + b140).
  Barrier from the perspective of persons with DB.
- Psychological health/mental health: people who were not working reported higher degree of anxiety, depression and suicidal thoughts and attempts (b198).
  Barrier from the perspective of person with DB.

Study 8:

- People with DB having additional physical disabilities (BODY FUNCTIONS, unspecified: additional disabilities).
  Barrier from the perspective of professional (variable associated with lower odds of competitive employment).

Study 11:

- Due to the progressive nature of Usher syndrome, participants had to face increasing limitations where adjustments made for deafblindness were no longer adequate. This leads to an unpredictable future regarding work (b210 + b230).
  Barrier from the perspective of persons with DB
- These limitations were increasing of psychical and mental symptoms such as: headaches, body pain and constant fatigue (BODY FUNCTIONS, unspecified: additional disabilities).
  Barrier from the perspective of person with DB

Study 12:

- Barriers due to Usher syndrome itself:
  - Influence on career choice;
  - Having to change career/location or to stop working;
  - Loss of productivity;
  - More efforts needed;
  - Not being able to do usual tasks;
  - Having to take days off due to Usher Syndrome. (b210 + b230).

  Barrier from the perspective of person with DB.

Study 13:

- Having dual sensory loss: participants with dual sensory are more likely to leave the labor force due to a disability (b210 + b230).] Although the relation was not significant after controlling for sex, ethnicity, number of health conditions and self-reported health.
  Barrier from the perspective of person with DB.

2. Activity and participation

2.1.1. Facilitators

Communication

Study 7:

- Slow communication with repetition and summarization from staff, entrepreneurs with DB and customers to ensure that the message is fully understood (d360 + e325).
  Communication techniques and suggestions.
- Another important communication issue is ensuring customers know the entrepreneur is deafblind. Alerting customers to the entrepreneur's dual sensory loss can smooth interactions (d360 + e345).
  Communication techniques and suggestions.

Self-care

Study 5:

- Physical health: persons in the working group had better physical health: less overweight, better at running short distance (b530 + d570).
  Facilitator from the perspective of person with DB.

Study 11:

- A balance between work and personal health needs to be found; in this study, participants tried to achieve this by taking on responsibility for recovery, accepting the need for adaptations, prioritizing work tasks and adding great effort/every effort possible (d598).
  Facilitator from the perspective of person with DB.

Interpersonal interactions and relationships

Study 3:

- Not disclosing they had Usher syndrome until after they had been hired (d720).
  Facilitator from the perspective of persons with DB.

Major life areas

Study 1:

- Volunteer work in integrated settings as preparation for paid work and building a resume (d855).
  Facilitator from the perspective of persons with DB

Study 2:

- Education level: having any kind of degree (including an associate degree up to a Ph.D. or professional degree) (d830).
  Facilitator from the perspective of person with DB
- Changing jobs or kind of work: changing the kind of work the person does changing their jobs because of their sensory loss (d845).
  Facilitator from the perspective of person with DB

Study 3:

- People with DB take the initiative to independently contact employers (d845).
  Facilitator from the perspective of persons with DB

Study 6:

- Paid high school work experiences: whether the youth ever worked for pay while in high school. Work experience was associated with higher likelihood of post-high-school employment (d859).
  Facilitator from perspective of multiple groups (predictor of postschool employment)
- Vocational education as a regular part of high school curriculum for youth with DB: lead to better job match on skills and interested, leading to sustained employment (d825).
  Facilitator from perspective of researchers (clinical implication)
- Career education for youth with DB must begin at an early age so children with DB become aware of the existence of jobs and develop prerequisite skills needed for employment (d825).
  Facilitator from perspective of researchers (clinical implication)
- Vocational education services: Whether the youth received career counselling, help finding a job, job skill training, or vocational education. The odds of continuous employment were higher for

youth who received vocational education services. Almost all youth who obtained these services received them from their schools (d825 + e590).
Facilitator from perspective of multiple groups (predictor of continuous employment)

Study 8:

- People with DB having a higher level of education (d830).
  Facilitator from the perspective of professional (variable associated with higher odds of competitive employment)
- People with DB obtaining a degree or certificate while receiving VR services (d839).
  Facilitator from the perspective of professional (variable associated with higher odds of competitive employment)
- For people with DB without an intellectual disability: Those who were employed at the application had much higher odds of competitive employment at closure (d859).
  Facilitator from the perspective of professional (variable associated with higher odds of competitive employment)
- People with DB having a higher level of education (d830).
  Facilitator from the perspective of professional (variable predictors of higher job quality)

Study 9:

- People with DB having college or university training (d830).
  Facilitator from the perspective of persons with DB

2.1.2. Barriers

Communication

Study 7:

- The most commonly cited challenge among BEP staff was ensuring the entrepreneurs with DB were communicating effectively with customers (d399).
  Barrier from the perspective of BEP staff.
- Staff also expressed concern about whether entrepreneurs with DB were correctly comprehending (receiving) content and lamented the difficulty of conveying (producing) lengthy or technical information (d329 + d349).
  Barrier from the perspective of BEP staff.
- Staff reported that communication with the deafblind entrepreneurs is characterized by repetition and slowness (d360 + e325)
  Barrier from the perspective of BEP staff.
- Other major challenges reported by both BEP staff and entrepreneurs with DB centred on the mechanics of communication, such as the difficulty of having one-on-one conversations and lack of spontaneity. In addition, both staff and entrepreneurs cited the fact that communication with deafblind entrepreneurs tends to take longer as a major challenge (d399).
  Barrier from the perspective of BEP staff and persons with DB.
- The entrepreneurs with DB themselves frequently reported having to ask customers to repeat themselves in order to correctly understand what they were saying (d310).
  Barrier from the perspective of persons with DB.

Mobility

Study 2:

- Not having transportation (d489).
  Barrier from the perspective of person with DB.

Interpersonal interactions and relationships

Study 1:

- Failure to achieve social integration in the organization: Social and communication interactions: job terminations are often related to social factors rather than poor performance of discrete job tasks (d740).
  Barrier from the perspective of persons with DB.

Major life areas

Study 2:

- Lack of training for a job (d825).
  Barrier from perspective of person with DB
- Having no college degree (d830).
  Barrier from perspective of person with DB
- Less like to change the kind of work the person does and changing jobs because of sensory loss (d845).
  Barrier from perspective of person with DB

Study 9:

- People with DB receiving disability-related augmentative skills training; this training is meant for visually impaired people and doesn't address the needs of people with DB. Ten services did not have significant relationships with competitive employment. These services were (a) basic academic remedial or literacy training, (b) job readiness training, (c) maintenance, (d) miscellaneous training, (e) on-the-job training, (f) occupational or vocational training, (g) rehabilitation technology, (h) reader services, (i) transportation, and (j) vocational rehabilitation guidance and training. (d825 + e585)
  Negative relationships with competitive employment outcomes

3. Environmental factors

3.1.1. Facilitators

Services, systems and policies

Study 1:

- Heterogeneous group placement (model): a job coach permanently supervises a number of workers with disabilities at a worksite. Dispersed heterogeneous groups: individuals with disabilities are dispersed throughout a central site, reflects the principle of natural proportion, enabling integration and social interaction with non-disabled coworkers. Heterogenous grouping, or the grouping of persons with differing degrees and types of disabilities within one placement requires less intensive supervision enables instructional support staff to maximize training for both individuals. A dispersed heterogenous group placement strategy in fact encompasses both of these issues: that of dispersal, which enhances integration and integration opportunities, and that of heterogeneity or dispersal among group members. Creation of an effective heterogeneous group requires attention to both the diversified skills and preferences of each group member (individualized job matching) and the diversified needs of the employer (e590).
  Facilitator from the perspective of persons with DB
- Job restructuring: Task redesign and provision of adaptations are strategies for promoting independence. Job restructuring extends the concept of adaptation beyond the individual tasks to the relationship among tasks. Restructured jobs are created by assigning one task currently performed by many different individuals to the target employee's position, or by recombining elements of several different job description into new jobs that utilize the specific job skills of the target employee. It may avoid the need for more costly adaptations such as continuous personal assistance (e590).
  Facilitator from the perspective of persons with DB
- Job sharing: a job that has traditionally been performed by one employee may be restructured into two or more parts, each performed by a different employee with disabilities (e590).
  Facilitator from the perspective of persons with DB
- Social skill training: systematic instruction in how to respond to social initiations and brief session between the employment specialist and selected coworkers on way to include the coworker with disabilities. Provision of systematic social skills training using behavioral technology (e585).
  Facilitator from the perspective of persons with DB

Study 2:

- Receiving help from their state VR agency/counselor or other agency to get or to keep a job (e590).
  Facilitator from the perspective of persons with DB

Study 3:

- VR-counselors with sign language skills, knowledge of progressive vision loss, and familiarity with DB (e590).
  Facilitator from the perspective of persons with DB
- VR-counselors that assist by contacting potential employers, providing support services for interviews (e590).
  Facilitator from the perspective of persons with DB
- VR-counselors communicate with person with DB instead of with the interpreter (e590).
  Facilitator from the perspective of persons with DB
- Pre-service education and in-service professional training for VR-counselors on the varied phenotypes of Usher syndrome and rehabilitation options (e585).
  Facilitator from the perspective of persons with DB
- Individual involvement (e.g. being involved in writing the individual plan for employment) in the rehabilitation process and counselling within VR (e590).
  Facilitator from the perspective of persons with DB
- VR-counselors that believe people with Usher can succeed professionally and should not convey lowered life or employment expectations (e455 + e590).
  Facilitator from the perspective of persons with DB

Study 4:

- Employment rehabilitation, such as retraining and employment counseling is important to individual with Usher (e590).
  Facilitator from the perspective of the researchers (clinical implications)

Study 5:

- Early interventions supporting by means of vocational training and other work-promoting activities instead of granting a disability pension (e590).
  Facilitator from the perspective of persons with DB
- All professionals in the field of rehabilitation should aways include work activity as a key element of interventions. VR-counselors/all professionals in the field of rehabilitation should aim at obtaining employment and remaining professionally active for as long as possible (e590).
  Facilitator from the perspective of persons with DB

Study 6:

- Professionals should educate parents early on (for example in early elementary school) about the employment possibilities for their child (e585).
  Facilitator from perspective of researchers (clinical implication)
- Vocational education services: Whether the youth received career counseling, help finding a job, job skill training, or vocational education. The odds of continuous employment were higher for youth who received vocational education services. Almost all youth who obtained these services received them from their schools (d825 + e590).
  Facilitator from perspective of multiple groups (predictor of continuous employment)

Study 7:

- Providing training in sign language or tactile signing techniques for BEP employees (e585).
  Communication techniques and suggestions
- Strengthen the BEP's partnerships with external organizations with expertise on DB to expand the resources for the entrepreneurs with DB (e598).
  Communication techniques and suggestions
- BEP staff felt it would be helpful if they had more knowledge about deafblindness (e585).
  Facilitator from the perspective of BEP staff
- Creating a set of guidelines for companies, including procedures for hiring qualified interpreters, outlining promising communication strategies, and addressing the unique technology needs of deafblind individuals (e590).
- *Facilitator from the perspective of BEP staff*

Study 8:

- People with DB receiving job placement assistance (e590).
  Facilitator from the perspective of professional (variable associated with higher odds of competitive employment)
- People with DB receiving job search assistance (e590).
  Facilitator from the perspective of professional (variable associated with higher odds of competitive employment)
- People with DB receiving short-term on-the-job-support or supported employment (e590).
  Facilitator from the perspective of professional (variable associated with higher odds of competitive employment)
- People with DB receiving VR counseling and guidance (e590).
  Facilitator from the perspective of professional (variable associated with higher odds of competitive employment

Study 9:

- People with DB receiving job-search assistance (e590).
  Facilitator from the perspective of persons with DB
- People with DB receiving job-placement assistance (e590).
  Facilitator from the perspective of persons with DB

Study 10:

- Consumers with DB served by agencies utilizing the specialist model or the professionals collaboration model or a combination were more likely to achieve competitive employment. No significant differences between these service models were found (e590).
  Facilitator from the perspective of professional (Service models)
- Consumers with DB served by blind agencies were more likely to achieve competitive employment, regardless of service model used (e590).
  Facilitator from the perspective of professional (Agency type)
- Agencies with specialized personnel served a larger percentage of consumers who are deafblind compared to agencies utilizing other service models. Specialized personnel understand the diverse characteristics, service needs, culture, and communication preferences of individuals who are deafblind; the specialized knowledge and skills of these professionals are invaluable in supporting deafblind consumers in achieving their employment goals. Having specialized personnel may lead to an increase in the number of deafblind consumers served and the quality of services (e590).
  Facilitator from the perspective of professional (Agency type)

Support and relationships

Study 1:
- Natural support: natural supports that are independent of paid agency personnel. The employment specialist provided training to informed co-workers and supervisors as adaptations and changes in the usual procedures were made. Co-workers developed their own strategies for teaching Lizbeth new tasks, and the company. Co-workers provide natural support (e325).
  Facilitator from the perspective of persons with DB

Study 3:
- Being inspired by the successes of other people with Usher syndrome: individuals who had acquired and maintained employment (e325).
  Facilitator from the perspective of persons with DB.

Study 6:
- Parents should be involved in specialized instruction, as youth who have skills in these key areas (self-care, communication, independent travel, computer use, receipt of regular diploma) may be more likely to have the basic skills needed to work (e310).
  Facilitator from perspective of researchers (clinical implication)
- Employed people with DB as role models to facilitate high expectations for both parents and child (e325).
  Facilitator from perspective of researchers (clinical implication)

Study 7:
- Slow communication with repetition and summarization from staff, entrepreneurs with DB and customers to ensure that the message is fully understood (d360 + e325).
  Communication techniques and suggestions
- Another important communication issue is ensuring customers know the entrepreneur is deafblind. Alerting customers to the entrepreneur's dual sensory loss can smooth interactions (d360 + e345).
  Communication techniques and suggestions
- Having access (both staff and entrepreneurs with DB) to interpreters and/or colleagues with knowledge of sign language or tactile interpreting. (e325 + e360).
  Communication techniques and suggestions
- Employing tactile interpreters as BEP employees. (e325 + e360).
  Communication techniques and suggestions

Study 11:
- Managers and colleagues who ask the person with deafblindness how to best adjust the workplace setting to facilitate active participation (e325 + e330)
  Facilitator from the perspective of persons with DB

Products and technology
Study 1:

- Augmentative and alternative communication systems: Provision of resources needed to enable employees to participate and communicate in the social context of the workplace, however, is an essential feature of integrated work models (e125).
  Facilitator from the perspective of persons with DB

Study 2:

- Assistive technology (AT) associated with their sensory loss at work (low vision aids, speech output devices, special lighting, special devices to use the telephone, braille devices). (e125 + e135).
  Facilitator from the perspective of person with DB

Study 4:

- To retain employment, individuals with Usher syndrome need to be evaluated for universal design and be equipped with assistive technology and other workplace adaptations (e135 + e299).
  Facilitator from the perspective of the researchers (clinical implications)

Study 7:

- The use of some form of hearing aid, cochlear implant, or personal sound amplifier while on the job (e125).
  Communication techniques and suggestions
- The use of accommodations including braille items, such as braille notetakers, braille labels, or braille displays; TTY telephones, laptops that customers can use to communicate with the entrepreneurs (e125).
  Communication techniques and suggestions
- Expanding and updating the technology available to BEP entrepreneurs with DB (e135).
  Communication techniques and suggestions

Attitudes
Study 2:

- Working within the blindness of deafness rehabilitation field: more availability and possibility of accommodations to the sensory loss (e498).
  Facilitator from the perspective of person with DB

Study 3:

- VR-counsellors that believe people with Usher can succeed professionally and should not convey lowered life or employment expectations (e455 + e590).
  Facilitator from the perspective of persons with DB

Study 6:

- Parent expectations: indicating the youth definitely won’t get a job at all until indicating the youth definitely will obtain a job an definitely will be self-supporting. Parent expectations were associated with higher likelihood of post-high school employment (e410).
  Facilitator from perspective of multiple groups (predictor of postschool employment)
- Parent expectations: indicating the youth definitely won’t get a job at all until indicating the youth definitely will obtain a job an definitely will be self-supporting. Parent expectation were associated with higher likelihood of continues employment. Parent expectations is especially important for youth with multiple disabilities (e410).
  Facilitator from perspective of multiple groups (predictor of continuous employment)

Study 7:

- Staff and entrepreneurs with DB stated that they felt that deafblindness did not create any unique challenges (e455 + PERSONAL FACTOR: Personal attitude).
  Perspective of both BEP staff and persons with DB

Study 11:

- Managers and colleagues who recognize that the person with deafblindness is a competent member of the workforce despite the difficulties caused by the deafblindness. (e425 + e430)
  Facilitator from the perspective of persons with DB

Natural Environment and human-made-changes to environment
Study 2:
- Accommodations at work related to their hearing or vision loss (e299).
  Facilitator from the perspective of person with DB

Study 4:
- To retain employment, individuals with Usher syndrome need to be evaluated for universal design and be equipped with assistive technology and other workplace adaptations (e135 + e299).
  Facilitator from the perspective of the researchers (clinical implications)

3.1.2. Barriers
Services, systems and policies
Study 3:
- VR-counselor with lowered employment expectations of people with DB (e455 + e590).
  Barrier from the perspective of persons with DB
- No assistance from VR-counselors in securing job interviews or support services for the job interviews: VR-counselor only provided computer links to employment listings and did not contact potential employers, did not offer assistance in securing interviews, and did not arrange or pay for support services for interviews (e590).
  Barrier from the perspective of persons with DB
- Many different VR-counselors during the rehabilitation process (e590).
  Barrier from the perspective of persons with DB

Study 5:
- People receiving 100% disability pension don't quality for unemployment benefits or rehabilitation. A consequence of this is that once a person has a disability pension, the change of returning to working life is low (e570).
  Barrier from the perspective of persons with DB
- Receiving 100% disability pension might enhance loss of confidence and feelings of worthlessness (e570).
  Barrier from the perspective of persons with DB

Study 8:
- Agency type of VR-service: odds were lower for those served by blind agencies compared to those who were served by combined agencies (e590).
  Barrier from perspective of professional (variable associated with lower odds of competitive employment)
- People with DB having a receipt of supplemental security income (SSI) (e570).
  Barrier from perspective of professional (predictor of lower job quality)
- People with DB having a receipt of social security disability insurance (SSDI) (e570).
  Barrier from perspective of professional (predictor of lower job quality)
- People with DB receiving short-term on-the-job-support or supported employment (e590).
  Barrier from perspective of professional (predictor of lower job quality)

Study 9:
- People with DB receiving disability-related augmentative skills training; this training is meant for visually impaired people and doesn't address the needs of people with DB. Ten services did not have significant relationships with competitive employment. These services were (a) basic academic remedial or literacy training, (b) job readiness training, (c) maintenance, (d) miscellaneous training, (e) on-the-job training, (f) occupational or vocational training, (g) rehabilitation technology, (h) reader services, (i) transportation, and (j) vocational rehabilitation guidance and training. (2 ICF codes: major life areas & services, systems and policies) (d825 + e585).
  Barrier from the perspective of persons with DB

Study 10:
- Consumers with DB served by agencies utilizing a miscellaneous service model were less likely to achieve competitive employment (e590).
  Barrier from perspective of professional (Service models)

- Consumers served by combined agencies were less likely to achieve competitive employment, regardless of service model used (e590).
  Barrier from perspective of professional (Agency types)

Attitudes
Study 2:
- Experiencing discrimination at work from their boss or employer (ever been denied a job or fired, laid off, or forces to resign from a job due to their sensory loss) (e430).
  Barrier from the perspective of person with DB
- Experiencing discrimination at work from their coworkers (e425).
  Barrier from the perspective of person with DB

Study 3:
- VR-counselor with lowered employment expectations of people with DB (e455 + e590).
  Barrier from the perspective of persons with DB
- Lack of consumer involvement in the rehabilitation process: professionals should regard people with Usher as participants in their rehabilitation planning and services. Being left out of conversations with the employer while being in the room (e455).
  Barrier from the perspective of persons with DB

Study 12:
- Negative reactions from colleagues (e425).
  Barrier from the perspective of person with DB

Products and technology
Study 1:
- Use of communication systems that violate the norms of conversational pragmatics because they require close proximity or sustained touching or fluency in a second language, may limit the degree to which the individual is perceiving as a participating member of the workplace (e125).
  Barrier from the perspective of persons with DB

Study 2:
- Less likely using assistive technology or lack of experience with assistive technology (e135).
  Barrier from the perspective of person with DB

Study 7:
- Relying on communication methods such as writing or speaking over the telephone, rather than speaking face to face (e125).
  Barrier from the perspective of both BEP staff and persons with DB
- Entrepreneurs expressed concern that the technology used by the BEP, such as talking calculators and hardcopy braillers, is out of date and that they lacked the tools necessary to fulfil their job roles successfully (e135).
  Barrier from the perspective of persons with DB

Support and relationships

Study 2:
- Being forced into retirement: For the national sample, a large majority reported being forced into retirement (e330)
  Barrier from the perspective of persons with DB

Study 7:
- Staff were frustrated by the difficulty of getting qualified interpreters to assist them in communicating with the entrepreneurs with DB (e360).
  Barrier from the perspectives of BEP staff.
- Staff reported that communication with the deafblind entrepreneurs is characterized by repetition and slowness (d360 + e325).
  Barrier from the perspectives of BEP staff.
- Reliance on outside help to communicate: When interacting with deafblind entrepreneurs, assistance was provided by professional interpreters, employees who know sign language or

fingerspelling, or family members. Rather than communicating primarily with the deafblind entrepreneur, some customers instead communicated with the entrepreneur's support staff. Such reliance on a third party for communication can pose challenges (e310 + e315 + e325 + e360). *Barrier from the perspective of both BEP staff and persons with DB*.

Natural Environment and human-made-changes to environment

Study 2:

- Less likely using accommodations at work (e299).
  Barrier from the perspective of person with DB

4. Personal factors

4.1.1. Facilitators

Study 4:

- Age: increased age the frequency of stress related to work decreased (being older) (PERSONAL FACTOR: age).
  Facilitator from the perspective of persons with DB
- Gender: women showed higher levels of stress than men (PERSONAL FACTOR: gender).
  Facilitator from the perspective of persons with DB

Study 5:

- Age: a difference was found in mean age between the working group (44 years) and the disability pension group (52 years) (PERSONAL FACTOR: age).
  Facilitator from the perspective of persons with DB.
- Early age of diagnosis could have positive effect on career planning (PERSONAL FACTOR: age of diagnosis).
  Facilitator from the perspective of persons with DB

Study 7:

- Staff and entrepreneurs with DB stated that they felt that deafblindness did not create any unique challenges (e455 + PERSONAL FACTOR: personal attitude).
  Perspective of both BEP staff and persons with DB

Study 8:

- People with DB who experience themselves as the primary source of support (PERSONAL FACTOR: self-reliance).
  Facilitator from perspective of professional (variable associated with higher odds of competitive employment)
- People with DB being younger of age (PERSONAL FACTOR: age).
  Facilitator from perspective of professional (predictor of higher job quality)
- People with DB who experience themselves as the primary source of support (PERSONAL FACTOR: self-reliance).
  Facilitator from perspective of professional (predictor of higher job quality)

4.1.2. Barriers

Study 2:

- The belief that older people with DB hold that employers think they are not able to work because of sensory loss (PERSONAL FACTOR: personal attitude about employers).
  Barrier from the perspective of person with DB
- The belief that older people with DB hold that they themselves think they are not able to work because of sensory loss (PERSONAL FACTOR: personal attitude about themselves).
  Barrier from the perspective of person with DB

Study 4:

- Age: with increased age the frequency of stress related to work decreased (being younger) (PERSONAL FACTOR: age).
  Barrier from the perspective of persons with DB
- Gender: Higher stress levels in women then in men on work overload, discontentment with work, being overwhelmed and chronic worry (PERSONAL FACTOR: gender).
  Barrier from the perspective of persons with DB

Study 5:

- Age: a difference was found in mean age between the working group (44 years) and the disability pension group (52 years) (PERSONAL FACTOR: age).
  Barrier from the perspective of persons with DB
- Late age of diagnosis: (1) chosen profession might be impossible to maintain with progressive visual impairment. (2) a delay in vocational training (3) long full-time sick leave after diagnosis causing difficulties returning to work (PERSONAL FACTOR: late age of diagnosis).

Barrier from the perspective of persons with DB

Study 8:

- Being female (PERSONAL FACTOR: gender).
  Barrier from perspective of professional (variable associated with lower odds of competitive employment)
- Being female (PERSONAL FACTOR: gender).
  Barrier from perspective of professional (predictor of lower job quality).
